# Ocular examinations, findings, and toxicity in children taking vigabatrin

**DOI:** 10.1016/j.jaapos.2022.05.001

**Published:** 2022-07-08

**Authors:** Yvette Schein, Keith D. Miller, Ying Han, Yinxi Yu, Alejandra G. de Alba Campomanes, Gil Binenbaum, Julius T. Oatts

**Affiliations:** a Division of Ophthalmology, Children’s Hospital of Philadelphia, Pennsylvania; b University of Michigan Medical School, Ann Arbor; c Department of Ophthalmology, University of California, San Francisco; d Scheie Eye Institute, Center for Preventive Ophthalmology and Biostatistics, Perelman School of Medicine, University of Pennsylvania, Philadelphia

## Abstract

**BACKGROUND:**

The antiepileptic medication vigabatrin has been associated with ocular toxicity, and close ophthalmic monitoring has been recommended; however, there is no clear consensus regarding the value and feasibility of such monitoring in children. We describe ophthalmic assessments in children in a real-world clinical setting, the incidence of vigabatrin-related ocular toxicity, and the utility of regular screening or ancillary testing in children taking vigabatrin.

**METHODS:**

The medical records of children taking vigabatrin with one or more ophthalmic assessments at Children’s Hospital of Philadelphia or University of California, San Francisco, between May 2010 and May 2021, were reviewed retrospectively. Abnormalities on ophthalmic examination, visual field (VF), electroretinogram (ERG), and optical coherence tomography (OCT) were reviewed and categorized as attributable to vigabatrin, possibly attributable to vigabatrin, or not attributable to vigabatrin.

**RESULTS:**

A total of 1,281 assessments of 284 children (mean age, 2.09 years) were included. Of these, 283 (99.6%) had funduscopic examination(s), 37 (13.0%) had ERG, 19 (6.7%) had OCT, and 6 (2.1%) had formal VF. Rate of examinations and ERGs per child decreased over the 10-year study period. Two children (0.7%) had definite vigabatrin-related ocular toxicity, both identified on ERG. An additional 4 children (1.4%) had optic atrophy of unclear relation to vigabatrin, categorized as possible toxicity. The remaining 278 children did not have abnormal examination or testing findings attributable to vigabatrin.

**CONCLUSIONS:**

The incidence of vigabatrin-related ocular toxicity in children was low in our cohort. Ocular and neurologic comorbidities and limited examinations in children make identification of such toxicity challenging and the value of screening is unclear.

Vigabatrin is an antiseizure medication used for the treatment of infantile spasms, simple or complex partial epilepsy, and other adult and childhood seizure disorders. Concerns about retinal or optic nerve toxicity associated with vigabatrin were first reported in adults in 1997; the extent to which vigabatrin causes clinically significant ocular pathology in children, however, is unknown.^1^ Vigabatrin is thought to cause permanent, concentric peripheral visual field loss in a dose-dependent manner.^2^ Additional findings of toxicity, primarily in adults, have included optic disk pallor and atrophy, retinal hypopigmentation, and macular wrinkling.^3–6^ Unfortunately, due to the young age and the high incidence of developmental delay in pediatric patients who need vigabatrin treatment, it is difficult for pediatric ophthalmologists to obtain information related to vigabatrin toxicity from standard ophthalmic examinations.^7,8^

The US Food and Drug Administration (FDA) recommends monitoring for retinal toxicity in patients receiving vigabatrin by performing an eye examination within 4 weeks of medication initiation and then every 3 months until the medication is stopped.^9^ Although the Vigabatrin Risk Evaluation and Mitigation Strategy program originally required frequent examinations, this requirement was subsequently changed to a recommendation.^10^ Additional testing, including formal visual field testing, is recommended for those able to participate. Given that the majority of children using vigabatrin are unable to complete perimetry, the American Association for Pediatric Ophthalmology and Strabismus issued a statement outlining the problems associated with screening for retinal toxicity in this group and recommended additional testing, such as electroretinography (ERG) and optical coherence tomography (OCT).^11^ However, ERG is expensive, often requires sedation, and may produce findings that are difficult to interpret, limiting their clinical utility^12^; and OCT presents similar feasibility challenges in young children.

The purpose of this study was to determine the rate at which ocular toxicity is detected in children taking vigabatrin in a real-world screening setting and how frequently these children receive ophthalmic evaluations. Understanding the incidence of vigabatrin-related toxicity in the pediatric population and the utility of ancillary testing may inform treatment and practice patterns for ophthalmologists and neurologists and potentially reduce the number of ophthalmic examinations.

## Subjects and Methods

We conducted a retrospective cohort study of all children prescribed vigabatrin who underwent one or more ophthalmic examinations and/or testing procedures related to vigabatrin at Children’s Hospital of Philadelphia and University of California, San Francisco, between May 2010 and May 2021. The study was considered exempt by the Children’s Hospital of Philadelphia Institutional Review Board (IRB) and was approved by the University of California, San Francisco IRB. Informed consent was waived at both institutions. Research was conducted in compliance with the US Health Insurance Portability and Accountability Act of 1996.

Eligible children were prescribed vigabatrin and had at least one ophthalmic assessment after starting treatment. Ophthalmic assessments included dilated fundus examination, ERG, OCT, or formal visual field (VF; automated static perimetry). Fundus examinations were performed by pediatric ophthalmologists and pediatric neuroophthalmologists. Children were excluded if their first assessment occurred 6 months or more after discontinuation of vigabatrin. Patients with only one examination or test were included to accurately reflect the “real-world” follow-up rate of these children.

Data abstracted from the medical record included demographic information; vigabatrin treatment dates; indication for vigabatrin use; dates of all fundus, ERG, OCT, and VF examinations occurring after medication initiation; abnormal findings on ophthalmic examination including optic atrophy, abnormal retinal pigmentation, and retinal atrophy; and abnormal findings on ERG, OCT, or VF testing. All abnormal examination and testing results were reviewed individually by three pediatric ophthalmologists (GB, ADA, JO) to determine whether the abnormalities were attributable to medication-related toxicity, as evidenced by temporal association, increasing atrophy, and/or inability to attribute finding to another disease process. In the case of discrepancy among the three independent reviews, a formal group consensus was reached based on additional detailed case review. Based on consensus, each ocular abnormality was categorized as attributable to vigabatrin, possibly attributable to vigabatrin, or not attributable to vigabatrin. Criteria for definite vigabatrin toxicity included ERG findings of a-wave and/or b-wave depression, and/or fundus examination findings of increasing optic atrophy related temporally to vigabatrin initiation, and not possibly due to another known medical or ophthalmic condition. Criteria for possible vigabatrin toxicity included presence or progression of optic atrophy not known to pre-date vigabatrin initiation, but with possible alternative explanation such as genetic abnormality, perinatal brain injury, or hydrocephalus. Abnormal ERGs or cases of optic atrophy were considered not attributable to vigabatrin if ERG findings were inconsistent with documented patterns associated with vigabatrin toxicity or if a second ERG was normal, and if optic atrophy pre-dated vigabatrin initiation and was better explained by known medical or ophthalmic conditions.

## Analysis

Descriptive statistics were calculated for baseline characteristics using mean, standard deviation, median, and interquartile ranges. The primary outcome was the proportion of children with ophthalmic pathology attributable to vigabatrin toxicity. The 95% confidence interval was calculated using the Wilson method. Secondary outcomes included number and types of assessments, including fundus examinations, ERG, OCT, and VF overall and by year to assess for changes in clinical practice over time. To determine assessment frequency, follow-up intervals were calculated between assessments for each child. Because ancillary testing such as ERG and OCT were often completed shortly before or after a clinic visit, only assessments that were greater than two months apart were considered as separate events. If a patient had two or more visits within a 2-month period, the earliest date was used. All analyses were completed with Microsoft Excel version 16.52 and SAS v9.4 (SAS Institute Inc, Cary, NC).

## Results

Over the study period, 284 children were prescribed vigabatrin and underwent a total of 1,281 assessments, including examinations and testing (Table 1). Fifty-one percent of children were female and the most frequent indication for vigabatrin was seizures secondary to a known genetic abnormality (39%). The median age at initiation of vigabatrin was 1.0 years (Q1 0.6, Q3 2.1), with a median treatment duration over the study period of 14.8 months (Q1 7.1, Q3 41.4) ranging from 0.2 to 139.4 months. The average time between medication initiation and ophthalmic assessment was 4.6 ± 7.5 (standard deviation) months (range, 0–58 months. Fifty-four percent of children had their first eye examination exclusively to screen for vigabatrin toxicity, while the remainder were being followed by an ophthalmologist prior to vigabatrin initiation or had additional coincident ophthalmic problems.

The number and interval of assessments were calculated for each examination type between 2010 and 2021. Children received a mean of 4.3 ± 4.5 assessments related to vigabatrin during the entire study period (range, 1–31). The child that received 31 examinations was an outlier: a 3.5 year old child with TUBA1A mutations causing lissencephaly and infantile spasms managed on vigabatrin from 2009 to 2021, with examinations every 3–6 months over that period. With regard to assessment type, 1,148 retinal examinations were performed on 283 children, of which 44 had an abnormality; 49 ERGs were performed on 37 children (range, 1–5 per child), of which 13 were abnormal in 12 children; 23 OCTs were performed on 19 children (range, 1–4 per child), of which 3 were abnormal in 3 children; and 11 formal VF tests performed on 6 children (range, 1–5 per child), of which 6 were abnormal in 2 children. The number of fundus examinations per child decreased steadily from 2013 to 2021. The ERG rate was highest in 2010 and decreased over time, with a low and stable rate from 2016 through 2021. The rate of OCT began and remained low over time. On average, children were assessed every 8.4 ± 5.7 months (range, 1.3–36.7). The mean follow-up interval increased with each calendar year.

With regard to ocular abnormalities, 44 children (15.5%) had an abnormality on fundus examination; 12 (32.4%) had an abnormal ERG; 3 (15.8%) had an abnormal OCT; and 2 (33.3%) had an abnormal VF (Table 2). In total, 6 children (2.1%) demonstrated definite or possible vigabatrin-related ocular toxicity (2 and 4 children, resp.). Detailed characteristics on these children are provided in eSupplement 1 (available at jaapos.org). Both children with definite toxicity were diagnosed by the treating ophthlamologists as having vigabatrin toxicity based on ERG findings of delayed and reduced a-wave and b-wave rod photoreceptor responses, both considered to be secondary to vigabatrin by the treating ophthalmologists. Four children had optic atrophy of indeterminate cause identified on fundus examination and deemed possibly related to vigabatrin due to chronicity of findings and possible alternative explanations. There were no children diagnosed with definite vigabatrin toxicity based on fundus examination, and no children had vigabatrin toxicity or possible vigabatrin toxicity diagnosed on the basis of an abnormal OCT or VF test.

Most abnormal fundus or testing abnormalities were unrelated to vigabatrin (Table 2). Thirty-one children had retinal findings unrelated to vigabatrin. Ten had optic nerve hypoplasia, and 6 had another optic nerve abnormality, including edema, cupping, neuropathy, and coloboma. Ten abnormal ERGs were explained by underlying retinal pathology unrelated to vigabatrin, including peripheral vasculopathy following laser photocoagulation, septooptic dysplasia, retinitis pigmentosa, or miscellaneous reasons, such as nonspecific abnormalities not correlated to any examination findings, subsequent normal ERG, or attributed to the effects of sedation. There was no clear pattern of heightened clinical concern for toxicity in those children referred for ERG versus those that were not.

## Discussion

We found a low incidence of vigabatrin-related ocular toxicity among children undergoing funduscopic examinations and ancillary testing. Only 2 children (0.7%) had definite toxicity, both identified through ERG testing, and an additional 4 (1.4%) had optic atrophy possibly but not definitively related to vigabatrin toxity. It is unclear whether our findings accurately reflect the rate of vigabatrin-related toxicity, or the low rate is due to the low proportion of children undergoing ancillary testing. Even among those undergoing ERG testing, only 5.4% had definite toxicity. At our institutions, the primary reasons for children on vigabatrin undergoing ERG were as follows: anesthesia required for another procedure, resulting in a decision to perform ERG to screen for vigabatrin toxicity at the same time; for a known retinal pathology (eg, retinal dystrophy); and in a nonstandardized manner at the physician’s discretion, to assess for vigabatrin toxicity, with or without a clinical suspicion for toxicity. All children but one underwent retinal examinations, no toxicity was diagnosed based on retinal lesions, and there were only 4 cases of optic atrophy questionably related to vigabatrin. In contrast, only 13% of children underwent ERG testing, which was the method by which the 2 clear cases of toxicity in our cohort were discovered. Both of the children with ERG-identified medication toxicity had a normal fundus examination, and both remained on the medication despite identified ocular toxicity because of its efficacy in seizure control. Similarly, while no toxicity was identified by OCT abnormality or perimetry, the majority of children were not able to perform these tests.

There are numerous reports of vigabatrin-associated visual field loss (VAVFL) in adults; however, data in children are not as robust, and the few reported studies in children have been of limited scope.^1,2,6,13–21^ Duration of treatment may be an important consideration in children, because some investigators have found an association between toxicity and length of treatment, although others have suggested that toxicity may be age dependent, with younger children being more susceptible.^19–22^ Medication guidelines for infantile spasms suggest discontinuing vigabatrin if there is no improvement in seizure control in the first 2–4 weeks of treatment.^9,10^ Our results show lower rates of toxicity than previously reported, especially considering that over 50% of children in our cohort had a treatment duration >12 months. Riikonen and colleagues^19^ observed VAVFL among 34% of children undergoing formal perimetry in a duration-dependent manner, with VAVFL present in 9% of children on vigabatrin for <1 year, 30% of children on vigabatrin for 1–2 years, and 63% of children on vigabatrin for >2 years. The latter study was limited by a relatively small sample size and inclusion of onlyolder children capable of performing formal perimetry.

A variety of funduscopic findings have been associated with vigabatrin use, including thinning of the nasal retinal nerve fiber layer, termed “inverse optic atrophy,” retinal pigment epithelial changes in the macula, and a membranous appearance to the retina.^3–5,11,23,24^ Optic atrophy was identified in 41 children (14%) of our cohort, none of whom had this finding attributed to vigabatrin in their clinical notes, likely due to the high prevalence of structural brain abnormalities and genetic conditions known to cause optic atrophy. We performed a secondary review of all cases of optic atrophy and determined that 4 children had optic atrophy possibly attributable to vigabatrin. They were classified as possibly and not definitively attributable due to the patients’ complex medical and neurologic comorbidities, with multiple potential causes of atrophy or with poorly documented chronicity of findings.

We found similarly low rates of toxicity on ancillary testing, with only 2 children showing evidence of vigabatrin related toxicity on ERG and none with evidence of toxicity on OCT or perimetry. A small minority of children in our cohort underwent this additional testing. In studies of vigabatrin exposed children, common ERG changes include reduced oscillatory potentials, reduced b-wave amplitudes, and diminished 30 Hz cone flicker responses.^13,25^ However, there are conflicting opinions about the utility of assessing vigabatrin associated retinal toxicity with ERG.^12,26^ In a study of vigabatrin exposed children who underwent both ERG and perimetry, there was no significant association between abnormal ERG parameters and VF defects.^27^ In both adults and children taking vigabatrin, OCT has shown retinal nerve fiber layer (RNFL) thinning.^28^ In a study of 18 children on vigabatrin, higher cumulative doses of vigabatrin were associated with greater RNFL thinning in the nasal, superior, and inferior quadrants.^29^ Our results support the idea that there is limited utility in such testing: only 2 children were found to have toxicity, and neither one discontinued vigabatrin because of those findings. In each case, the parents and physicians felt that the seizure-reducing benefits of continuing treatment outweighed the impact of ocular toxicity.

Our results reflect a practical or “real world” inspection of vigabatrin screening and highlight the heterogeneity of practice patterns and difficulties of assessing ocular toxicity in children. Because VF loss is difficult to assess in children who cannot participate in formal perimetric testing, pediatric ophthalmologists rely on fundus examinations and ancillary testing, such as ERG or OCT, for screening, although such ancillary tests often require sedation or general anesthesia, which is not without risk.^30^ While sedation may not be universally required for ERGs, the majority of ERGs performed at our two institutions use sedation. Additionally, awake ERGs require technology that may not be widely available as well as a patient who can tolerate the procedure. The developmental status and comorbidities of many children taking vigabatrin make this impractical.

Although Westall and colleagues^22^ included structured ERG protocols to identify toxicity, our study suggests that the infrastructure required to support such an approach is not available to many pediatric ophthalmologists.

We found a wide variety in patient assessments, with no clear pattern of heightened concern in those children who were referred for ancillary testing. We also found that length of time between vigabatrin-related ocular assessments increased from 2010 to 2021. This may reflect a growing understanding among pediatric ophthalmologists of the low yield of fundus examinations for detecting toxicity, the elimination of FDA-required examinations, and ambiguous and heterogeneous definitions of presumed vigabatrin-related ocular toxicity. Even in cases of possible or definite toxicity, no children in our study stopped vigabatrin due to vision concerns. In many cases, an unwillingness to stop vigabatrin even in the setting of a hypothetical ERG abnormality was cited as the reason why ERG testing was not pursued. If toxicity is difficult to identify, there is heterogeneity in patient assessment, many children have limited visual potential due to comorbidities, and the results of testing for toxicity are unlikely to change management, there may not be much value in assessing patients who have not had a functional decline in vision. Additionally, a conversation with parents about whether findings of toxicity would change seizure management could help pediatric ophthalmologists determine whether following a given patient with serial fundus examinations or ancillary testing is warranted.

Strengths of our study include a large sample size from two tertiary referral centers and uniform methodology. However, there are several limitations to consider, including loss to follow-up, potentially different practice patterns by institution or provider, and the relatively small number of ERGs, OCTs, and formal VF tests performed. It is possible that loss to follow-up may contribute to the low incidence of vigabatrin toxicity in our population. We attempted to limit the effect of institutional differences by having pediatric ophthalmologists from both institutions concur on identification of cases of suspected or definite medication-related toxicity. Due to the small number of ancillary tests, we are unable make firm conclusions about the incidence of vigabatrin toxicity or the utility of these tests. However, our results add weight to the body of literature that questions the utility of these assessments in terms of financial costs, difficulty in identifying clinically meaningful toxicity, and lack of influence on clinical decision making.

In what we believe to be the largest study of vigabatrin-related ocular toxicity in children, we found that rates of toxicity were low in a real-world screening setting. Given the high degree of comorbid conditions that limit visual potential in this population and the clear clinical benefit of seizure control that many children receive from vigabatrin, discontinuation of vigabatrin may not be warranted even in the event of ocular toxicity. We found that there has been a decline in the number of retinal examinations, ERGs, and OCTs being performed at our institutions each year, reflecting growing recognition of the limited clinical benefit of regular screening examinations and testing for detecting vigabatrin-related ocular toxicity.

## Supplementary Material

supplemental

## Figures and Tables

**Table 1.** Baseline characteristics

Children with at least 1 fundus/OCT/VF/ERG assessment AFTER initiating vigabatrin	No.^a^

Sex	
Female	145/284 (51.1)
Indication for vigabatrin	
Seizures—systemic/genetic abnormalities	111 (39.1)
Tuberous sclerosis	49 (17.3)
Infantile spasms (not otherwise specified)	77 (27.1)
Seizures—structural brain abnormalities	39 (13.7)
Lennox-Gastaut syndrome	26 (9.2)
Hypoxic/perinatal brain injury	16 (5.6)
Epilepsy (not otherwise specified)	12 (4.2)
Other	3(1.1)
Age at initiation, years	
Mean ± SD	2.09 ± 2.9
Median (Q1, Q3)	1.0 (0.6, 2.1)
Range	0.1 to 16.0
Duration of treatment, months	
Mean ± SD	28.8 ± 33.0
Median (Q1, Q3)	14.8 (7.1,41.4)
Range	0.2 to 139.4
Time until first eye assessment (months)	
Mean ± SD	4.62 ± 7.54
Median (Q1, Q3)	2.07 (0.7, 4.7)
Range	0.00 to 58.0
First assessment exclusively for vigabatrin	154 (54.2)

*ERG*, electroretinogram; *OCT*, optical coherence tomography; *Q1*, first quartile; *Q3*, third quartile; *SD*, standard deviation; *VF*, visual field.

a Parenthetical values indicate percentage, except as noted.

**Table 2.** Examination findings

Study parameter	No. (%)

Fundus examinations	283
Abnormal fundus examinations (possibly related)	44 (15.5)
Not attributable to vigabatrin	40 (14.1)
Inconclusive	4(1.4)
Attributable to vigabatrin	0 (0)
ERG	37
Abnormal ERG	12 (32.4)
Not attributable to vigabatrin	10 (27.0)
Inconclusive	0 (0)
Attributable to vigabatrin	2 (5.4)
OCT	19
Abnormal OCT	3 (15.8)
Not attributable to vigabatrin	3 (15.8)
Inconclusive	0 (0)
Attributable to vigabatrin	0 (0)
Visual Fields	6
Abnormal Visual Fields	2 (33.3)
Not attributable to vigabatrin	2 (33.3)
Inconclusive	0(0)
Attributable to vigabatrin	0 (0)
Examination findings UNRELATED to vigabatrin	283
Cortical visual impairment	81 (28.6)
Strabismus	63 (22.3)
Non-medication-related retinal lesions^a^	31 (10.9)
Optic atrophy	37 (13.1)
Optic nerve hypoplasia	10 (3.5)
Other optic nerve	6(2.1)
VF deficit	9 (3.2)
Other^b^	38 (13.4)
No abnormality	37 (13.1)

*ERG*, electroretinogram; *OCT*, optical coherence tomography.

a Retinal changes included: lacunae, hamartoma, congenital hypertrophy of the retinal pigment epithelium (CHRPE), bear tracks, astrocytoma, laser scars, retinal detachment, fungal chorioretinitis, non-medication-related retinal pigment changes.

b Other included nystagmus, pigment, cataract, microphthalmos, nasolacrimal duct obstruction, corneal abrasion or ulcer, corneal scar or opacity, exposure keratopathy, anisocoria, retrolental membrane, subconjunctival hemorrhage, glaucoma, persistent fetal vasculature, iris hypopigmentation, iris coloboma.

## References

[1] Eke T, Talbot JF, Lawden MC. Severe persistent visual field constriction associated with vigabatrin. BMJ 1997;314:180–81. 9022432 10.1136/bmj.314.7075.180 PMC2125673

[2] Malmgren K, Ben-Menachem E, Frisen L. Vigabatrin visual toxicity: evolution and dose dependence. Epilepsia 2001;42:609–15. 11380567 10.1046/j.1528-1157.2001.28600.x

[3] Buncic JR, Westall CA, Panton CM, Munn JR, MacKeen LD, Logan WJ. Characteristic retinal atrophy with secondary “inverse” optic atrophy identifies vigabatrin toxicity in children. Ophthalmology 2004;111:1935–42. 15465561 10.1016/j.ophtha.2004.03.036 PMC3880364

[4] Koul R, Chacko A, Ganesh A, Bulusu S, Al RiyamiK. Vigabatrin associated retinal dysfunction in children with epilepsy. Arch Dis Child 2001;85:469–73. 11719329 10.1136/adc.85.6.469 PMC1719022

[5] Krauss GL, Johnson MA, Miller NR. Vigabatrin-associated retinal cone system dysfunction: electroretinogram and ophthalmologic findings. Neurology 1998;50:614–18. 9521245 10.1212/wnl.50.3.614

[6] Lawden MC, Eke T, Degg C, Harding GF, Wild JM. Visual field defects associated with vigabatrin therapy. J Neurol Neurosurg Psychiatry 1999;67:716–22. 10567485 10.1136/jnnp.67.6.716 PMC1736662

[7] Hancock EC, Osborne JP, Edwards SW. Treatment of infantile spasms. Cochrane Database Syst Rev 2013;6:CD001770. 10.1002/14651858.CD001770.pub3 PMC11194850 23740534

[8] Menacker SJ, Fernandes A, Ward L. Prevalence of visual impairment, ocular pathology, and ability to achieve a thorough examination in an eye clinic for patients with disabilities. J AAPOS 2019;23:274.e1–5. 10.1016/j.jaapos.2019.06.001 31513903

[9] Sabil: Highlights of Prescribing Information. Published online 2018. https://www.accessdata.fda.gov/drugsatfda_docs/label/2018/022006s020,020427s018lbl.pdf.

[10] Sabril Medication Guide. Published online January 2020. https://www.lundbeck.com/content/dam/lundbeck-com/americas/united-states/products/neurology/sabril_mg_us_en.pdf.

[11] Vigabatrin: The Problem of Monitoring for Peripheral Vision Loss in Children. Published online July 2017. https://aapos.org/HigherLogic/System/DownloadDocumentFile.ashx?DocumentFileKey5781a381d-6dae-ede4-bcf7-a3db9973bf15.

[12] Jastrzembski B, Locke J, Wan MJ. Clinical implications and cost of electroretinography screening for vigabatrin toxicity. Can J Ophthalmol 2020;55:e98–100. 31879070 10.1016/j.jcjo.2019.10.014

[13] Foroozan R Vigabatrin: lessons learned from the United States experience. J Neuro-Ophthalmol Off J NorthAm Neuro-Ophthalmol Soc 2018;38:442–50. 10.1097/WNO.0000000000000609 29280765

[14] Daneshvar H, Racette L, Coupland SG, Kertes PJ, Guberman A, Zackon D. Symptomatic and asymptomatic visual loss in patients taking vigabatrin. Ophthalmology 1999;106:1792–8. 10485552 10.1016/S0161-6420(99)90345-7

[15] Gaily E, Jonsson H, Lappi M. Visual fields at school-age in children treated with vigabatrin in infancy. Epilepsia 2009;50:206–16. 19215279 10.1111/j.1528-1167.2008.01961.x

[16] Iannetti P, Spalice A, Perla FM, Conicella E, Raucci U, Bizzarri B. Visual field constriction in children with epilepsy on vigabatrin treatment. Pediatrics 2000;106:838–42. 11015531 10.1542/peds.106.4.838

[17] Kälviäinen R, Nousiainen I, Mäntyjärvi M, Vigabatrin, a gabaergic antiepileptic drug, causes concentric visual field defects. Neurology 1999;53:922–6. 10496247 10.1212/wnl.53.5.922

[18] Maguire MJ, Hemming K, Wild JM, Hutton JL, Marson AG. Prevalence of visual field loss following exposure to vigabatrin therapy: a systematic review. Epilepsia 2010;51:2423–31. 21070215 10.1111/j.1528-1167.2010.02772.x

[19] Riikonen R, Rener-Primec Z, Carmant L, Does vigabatrin treatment for infantile spasms cause visual field defects? An international multicentre study. Dev Med Child Neurol 2015;57:60–67. 25145415 10.1111/dmcn.12573

[20] Vanhatalo S, Nousiainen I, Eriksson K, Visual field constriction in 91 Finnish children treated with vigabatrin. Epilepsia 2002;43:748–56. 12102679 10.1046/j.1528-1157.2002.17801.x

[21] Schwarz MD, Li M, Tsao J, A lack of clinically apparent vision loss among patients treated with vigabatrin with infantile spasms: The UCLA experience. Epilepsy Behav EB 2016;57(Pt A):29–33. 10.1016/j.yebeh.2016.01.012 26921595

[22] Westall CA, Wright T, Cortese F, Kumarappah A, Snead OC, Buncic JR. Vigabatrin retinal toxicity in children with infantile spasms: An observational cohort study. Neurology 2014;83: 2262–8. 25381295 10.1212/WNL.0000000000001069 PMC4277676

[23] Frisén L, Malmgren K. Characterization of vigabatrin-associated optic atrophy. Acta Ophthalmol Scand 2003;81:466–73. 14510793 10.1034/j.1600-0420.2003.00125.x

[24] Wild JM, Robson CR, Jones AL, Cunliffe IA, Smith PEM. Detecting vigabatrin toxicity by imaging of the retinal nerve fiber layer. Invest Ophthalmol Vis Sci 2006;47:917–24. 16505024 10.1167/iovs.05-0854

[25] Morong S, Westall CA, Nobile R, Longitudinal changes in photopic OPs occurring with vigabatrin treatment. Doc Ophthalmol Adv Ophthalmol 2003;107:289–97. 10.1023/b:doop.0000005338.51554.e3 PMC3880363 14711161

[26] Strong AD, Sturdy A, McCourt EA, Clinical utility of electroretinograms for evaluating vigabatrin toxicity in children. J Pediatr Ophthalmol Strabismus 2021;58:174–9. 34039156 10.3928/01913913-20210111-03

[27] Moskowitz A, Hansen RM, Eklund SE, Fulton AB. Electroretinographic (ERG) responses in pediatric patients using vigabatrin. Doc Ophthalmol Adv Ophthalmol 2012;124:197–209. 10.1007/s10633-012-9320-7 22426576

[28] Clayton L, Devile M, Punte T, Patterns of peripapillary retinal nerve fiber layer thinning in vigabatrin-exposed individuals. Ophthalmology 2012;119:2152–60. 22853973 10.1016/j.ophtha.2012.05.009

[29] Origlieri C, Geddie B, Karwoski B, Optical coherence tomography to monitor vigabatrin toxicity in children. J AAPOS 2016;20: 136–40. 27079594 10.1016/j.jaapos.2015.10.020

[30] Schneuer FJ, Bentley JP, Davidson AJ, The impact of general anesthesia on child development and school performance: a population-based study. Paediatr Anaesth 2018;28:528–36. 29701278 10.1111/pan.13390

