# Supplementary material for: Ocular examinations, findings, and toxicity in children taking vigabatrin
Source: J AAPOS. Author manuscript; Available in PMC 2024 Mar 18. (PMC10947413; doi:10.1016/j.jaapos.2022.05.001)
Supplement: supplemental [file NIHMS1971170-supplement-supplemental.pdf]

Schein Y, Miller KD, Han Y, Yu Y, de Alba Campomanes AG, Binenbaum G, Oatts JT. Ocular examinations, findings, and toxicity in children taking vigabatrin. J AAPOS online supplement.

eSupplement 1. Characteristics of six children with definite or possible vigabatrin toxicity

Patient 1	
Toxicity	DEFINITE
Indication for vigabatrin	Tuberous sclerosis, infantile spasms
Major comorbidities	Global developmental delay
Age at vigabatrin initiation, years	10.3
Vigabatrin duration, months	116.6
Total assessments	12
ERG details	• Had ERG because of reduced visual engagement concerns<br>• ERG showed b-wave depression<br>• Treating ophthalmologist attributed this to vigabatrin
Fundus details	• Normal at the time of ERG<br>• Pallor and vessels with slight attenuation, tortuosity 2 years later
Visual acuity	No fix, no follow either eye
Reason for determining toxicity	ERG with b-wave depression
Outcome	• Continued vigabatrin use despite ERG confirmed toxicity<br>• Family and medical team agreed seizure control outweighed consequence of possible vision loss
Patient 2	
Toxicity	DEFINITE
Indication for vigabatrin	Infantile spasms
Major comorbidities	Congenital cerebral malformations right > left
Age at vigabatrin initiation, years	0.8
Vigabatrin duration, months	19.3
Total assessments	2
ERG details	• Reduction of a-wave and b-wave amplitudes<br>• Treating ophthalmologist attributed this to vigabatrin
Fundus details	Normal Discs and retinas
Visual acuity	Fix and follow with both eyes (monocular testing not possible)
Reason for determining toxicity	Reduction of a-wave and b-wave amplitudes
Outcome	• Continued vigabatrin despite ERG-confirmed toxicity<br>• Family and medical team agreed seizure control outweighed consequence of possible vision loss
Patient 3	
Toxicity	INCONCLUSIVE
Indication for vigabatrin	Intractable focal epilepsy
Major comorbidities	Mitochondrial depletion syndrome, cerebellar atrophy
Age at vigabatrin initiation, years	3.7
Vigabatrin duration, months	125.2
Total assessments	27
ERG details	No ERG performed
Fundus details	• Initially had a normal fundus exam<br>• Over time developed mild optic atrophy, then moderate
Visual acuity	Fix and follow each eye
Reason for determining toxicity	Inconclusive because although the patient had worsening optic atrophy on exam, we could not rule out underlying cerebral atrophy as a cause
Outcome	Continued vigabatrin use despite optic atrophy; family and medical team agreed seizure control outweighed consequence of possible vision loss
Patient 4	
Toxicity	INCONCLUSIVE
Indication for vigabatrin	Infantile spasms
Major comorbidities	Chromosomal deletion in chromosome 16
Age at vigabatrin initiation, years	0.9
Vigabatrin duration, months	10.5
Total assessments	3
ERG details	• No ERG performed<br>• Documented conversation: "discussed ERG testing for vigabatrin, which would be difficult and likely abnormal due to vision impairment and difficult to interpret. Would only consider if parents are seeing changes in visual function or we see retinal changes over time”

Copyright 2022 by the American Association for Pediatric Ophthalmology and Strabismus

Fundus details	2+ optic atrophy in both eyes
Visual acuity	CUSM each eye
Reason for determining toxicity	Inconclusive because although the patient had optic atrophy on exam after initiating vigabatrin, we could not rule out underlying comorbidities as a cause of this finding. The treating ophthalmologist thought the atrophy was likely related to central visual pathway abnormality
Outcome	Continued vigabatrin use despite optic atrophy; family and medical team agreed seizure control outweighed consequence of possible vision loss
Patient 5	
Toxicity	INCONCLUSIVE
Indication for vigabatrin	Perinatal brain injury causing seizures
Major relevant comorbidities	Prematurity with history of cerebral hemorrhage, periventricular leukomalacia, severe white matter volume loss with associated colpocephaly
Age at vigabatrin initiation, years	0.7
Vigabatrin duration, months	25.5
Total ophthalmic assessments	7
ERG details	No ERG performed
Fundus details	Slight optic pallor and cupping in both eyes
Visual acuity	UCUSUM each eye, no response to Teller cards
Reason for determining toxicity	Inconclusive because although the patient had optic pallor on exam after initiating vigabatrin, we could not rule out underlying comorbidities as a cause of this finding
Outcome	Continued vigabatrin use despite optic atrophy; family and medical team agreed seizure control outweighed consequence of possible vision loss
Patient 6	
Toxicity	INCONCLUSIVE
Indication for vigabatrin	Tuberous sclerosis
Major relevant comorbidities	Scattered cortical tubers, worst in posterior lobes
Age at vigabatrin initiation, years	4.3
Vigabatrin duration, months	21.1
Total ophthalmic assessments	1
ERG details	No ERG performed
Fundus details	1+ optic nerve pallor in both eyes
Visual acuity	20/40, 20/30 by LEA matching
Reason for determining toxicity	Inconclusive because although the patient had optic pallor on exam after initiating vigabatrin, we could not rule out underlying comorbidities as a cause of this finding
Outcome	Continued vigabatrin use despite optic atrophy; family and medical team agreed seizure control outweighed consequence of possible vision loss

CUSM, central unsteady maintained; *ERG*, electroretinogram; *UCUSUM*, uncentral unsteady unmaintained.

Copyright 2022 by the American Association for Pediatric Ophthalmology and Strabismus
